# Post‐implant computed tomography–magnetic resonance prostate image registration using feature line parallelization and normalized mutual information

**DOI:** 10.1120/jacmp.v8i1.2351

**Published:** 2007-02-28

**Authors:** Sandra Vidakovic, Hans S. Jans, Abe Alexander, Ron S. Sloboda

**Affiliations:** ^1^ Cross Cancer Institute Department of Medical Physics Edmonton Alberta Canada; ^2^ Cross Cancer Institute Department of Radiation Oncology Edmonton Alberta Canada; ^3^ University of Alberta Department of Oncology Edmonton Alberta Canada

**Keywords:** prostate brachytherapy, CT–MR image registration, normalized mutual information, post‐implant dosimetry

## Abstract

Post‐implant dosimetry for permanent prostate brachytherapy is typically performed using computed tomography (CT) images, for which the clear visualization of soft tissue structures is problematic. Registration of CT and magnetic resonance (MR) image volumes can improve the definition of all structures of interest (soft tissues, bones, and seeds) in the joint image set. In the present paper, we describe a novel two‐stage rigid‐body registration algorithm that consists of (1) parallelization of straight lines fit to image features running primarily in the superior–inferior (Z) direction, followed by (2) normalized mutual information registration. The first stage serves to fix rotation angles about the anterior–posterior (Y) and left–right (X) directions, and the second stage determines the remaining Z‐axis rotation angle and the X, Y, Z translation values. The new algorithm was applied to CT and 1.5T MR (T2‐weighted and balanced fast‐field echo sequences) axial image sets for three patients acquired four weeks after prostate brachytherapy using I125 seeds. Image features used for the stage 1 parallelization were seed trains in CT and needle tracks and seed voids in MR. Simulated datasets were also created to further investigate algorithm performance. Clinical image volumes were successfully registered using the two‐stage approach to within a root‐mean‐squares (RMS) distance of <1.5 mm, provided that some pubic bone and anterior rectum were included in the registration volume of interest and that no motion artifact was apparent. This level of accuracy is comparable to that obtained for the same clinical datasets using the Procrustes algorithm. Unlike Procrustes, the new algorithm can be almost fully automated, and hence we conclude that its further development for application in post‐implant dosimetry is warranted.

PACS numbers: 87.53.Jw, 87.57.Gg, 87.59.Fm, 87.61.Pk

## I. INTRODUCTION

Post‐implant dosimetry for permanent prostate implants yields an estimate of the dose distribution delivered to the patient based on measured, rather than planned, radioactive source positions. To achieve meaningful post‐implant dosimetry analysis, the sources must be clearly identifiable and their positions within the patient's anatomy relative to the target volume and critical structures must be known precisely. Therefore, it is crucial to delineate structure contours and determine source locations accurately.

In current practice, post‐dosimetry is most often performed using computed tomography (CT) images, and calculations of dosimetric indices are based on the TG‐43 dose calculation formalism.^(1)^ Although CT images provide excellent visualization of the seed sources, soft tissues are often poorly differentiated, which admits subjective judgment into delineation of the target contour. In general, the prostate volume is overestimated,^(^ ^2^ ^,^ ^3^ ^)^ leading to inaccurate values for dosimetric indices,^(^ ^4^ ^,^ ^5^ ^)^ which in turn can affect the assessment of implant quality. Conversely, anatomic structures are well defined in T2‐weighted MR images^(6)^; however, distinguishing between signal voids associated with seeds and blood vessels in these images is difficult.^(7)^ Neither imaging modality provides clear visualization of both the seeds and the anatomy, and therefore neither CT nor MR images alone are fully adequate for post‐implant dosimetry.

Registration of CT and MR datasets combines the information from the two modalities and has the potential to provide accurate visualization in a joint image set of prostate volume, organs at risk, and seeds. Researchers have previously used various methods to attempt registration of CT–MR prostate post‐implant datasets. These methods have included matching anatomic landmarks such as bones^(^ ^8^ ^,^ ^9^ ^)^ and bladder base and urethra.^(10)^ Others have involved manually matching seeds^(^ ^9^ ^,^ ^11^ ^)^ and, more recently, using a mutual information technique.^(^ ^12^ ^,^ ^13^ ^)^ Prostate mobility with respect to other anatomic structures introduces problems into landmark‐based registration,^(9)^ but manual seed matching is a time‐consuming process that requires identification of corresponding seed pairs in the CT and MR datasets.^(13)^ By comparison, registration based on the mutual information technique is a largely automated procedure that makes efficient use of a relatively large amount of image data.

In their 2004 paper, McLaughlin et al.^(12)^ reported on an MR‐axial to CT‐axial registration process based on mutual information and a three‐dimensional (3D) rigid‐body transformation (three rotations and three translations). Successful registration, with an average overall uncertainty of 1.4 mm, was consistently achieved only by cropping the MR images in such a way that the volume of interest included minimal pubic bone anteriorly and some rectum posteriorly. They found that “prostate‐only registration did not result in a successful end point because the information in the prostate was not sufficient to prevent large rotation angles.”

As mentioned, the position of the prostate relative to adjacent anatomic structures such as bones and rectum may be different in the CT and MR image sets considering the time interval between the scans (allowing rectal and bladder filling) and the difference in scan environments (different couches, presence of pelvic coil).^(^ ^14^ ^,^ ^15^ ^)^ Consequently, the ability to perform prostate‐only registration would be clinically advantageous.

In the present paper, we describe a novel hybrid registration method that makes use of a registration volume including only prostate and immediately surrounding tissue. Registration is performed in two stages. The first stage involves obtaining a transformation to parallelize straight lines fit to corresponding features running primarily in the superior–inferior (Z) direction in the CT and MR image volumes. This initial transformation serves to establish relative rotation angles in the lower information density sagittal (Y–Z) and coronal (X–Z) image planes. The second stage consists of applying a normalized mutual information (NMI) algorithm (Analyze v5.0 software: AnalyzeDirect, Lenexa, KS) to obtain the remaining relative X, Y, and Z translations and the Z‐axis rotation required to complete the 3D rigid‐body registration process. Limiting rotational degrees of freedom (DF) to rotation about the Z‐axis for only NMI is expected to provide a better opportunity to achieve prostate‐only registration of CT and MR volume sets. Application of the new algorithm is illustrated here for three clinical cases. Assessment of its performance within the context of a prospective clinical study currently in progress will be reported separately.

## II. METHODS

Initial attempts to use the NMI algorithm available in Analyze 5.0 software to register clinical CT and MR datasets with a rigid‐body transformation and allowing 6 DF proved to be unreliable. To study the problem further, we used Matlab v7.1 code (The MathWorks, Natick, MA) to build simulated CT and MR 3D image volumes of an implant so that we could directly manipulate image content and features. Moreover, considering that it is possible to observe the positions of seeds (CT) and needle tracks (MR) in the axial slices of post‐implant clinical images, we postulated that it may be possible to determine relative rotations about the X and Y imaging axes by independent means before NMI registration. Knowing those angles would eliminate two rotational DF, which are associated with the lower‐information‐density sagittal (Y–Z) and coronal (X–Z) image planes from the NMI registration process.

Our proposed approach to this task is the feature lines method, wherein a clearly identifiable seed train and needle track is represented as a straight line along the average direction of the train and track. Such a line can be obtained as the best fit, in 3D space, to seed and needle track positions extracted from the axial slices comprising CT and MR image volumes respectively. Determining the transformation that parallelizes a corresponding pair of feature lines yields the required relative rotations about the X and Y imaging axes.

## A. Clinical datasets

Clinical CT and MR image volumes for three patients were acquired approximately 4 weeks post‐implant using a 15.0 cm field of view. The CT volume set was acquired using a Picker PQ‐5000 scanner (Philips Medical Systems, Bothell, WA), applying a standard non‐helical scan with 3.0‐mm slice thickness and ~0.3‐mm pixel pitch. Two different pulse sequences were used to acquire a set of axial MR images^(16)^: a T2‐weighted sequence with TE/TR=91/4748 ms and a balanced fast‐field echo (B‐FFE)^(16)^ sequence with TE/TR=9.6/4.8 ms. Both MR volumes were obtained using a Philips Gyroscan Intera 1.5T MRI system (Philips Medical Systems) with a surface coil (Sense cardiac). In each case, axial slices were 3 mm thick, with no gap between them, and pixel pitches were again ~0.3 mm.

**Figure 1:**
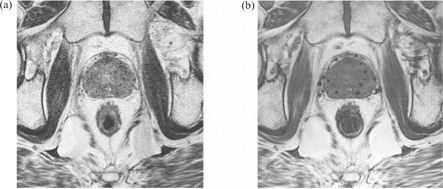
(a) Axial slice of T2‐weighted magnetic resonance (MR) volume set of post‐implant prostate. (b) Approximately corresponding axial slice of balanced fast‐field echo (B‐FFE) MR volume.

As illustrated in Fig. 1, a B‐FFE image provides better visualization of needle tracks and is less noisy than a T2‐weighted image, in which needle tracks are hard to distinguish from blood vessels and image noise. We therefore decided to use B‐FFE MR volumes in our analysis. To minimize prostate movement between the two scans, CT and MR image volumes were acquired under similar clinical conditions about 2 hours apart. Patients were asked to empty their bladder before each scan, and they were imaged supine with an under‐knee rest providing support to facilitate reproducible positioning.

## B. Simulated datasets

**Figure 2:**
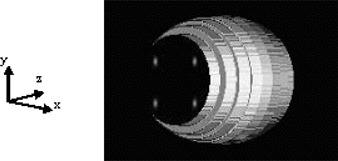
Volume rendering of a simulated computed tomography (CT) prostate implant dataset.

The simulated datasets consisted of 13 axial slices, each 3 mm thick, containing a geometric solid prostate (~32 cc ellipsoid) centered in the volume, a uniform background, and blurred images of seeds (CT) or seed voids (MR). A total of 20 simulated seeds or seed voids were grouped into four trains (five seeds per train), each running parallel to the Z‐axis, with seed lengths also oriented in the Z direction. The voxel dimensions of our simulated datasets, 0.29×0.29×3.0 mm, corresponded to those of the clinical images. The intensities (pixel values) of objects in the simulated datasets were selected to correspond approximately to the average intensities of the same objects in the clinical datasets, and are given in Table 1. Poisson noise was optionally included. Fig. 2 shows a volume rendering of a simulated CT dataset.

**Table 1:** Structure intensities (pixel values) in simulated computed tomography (CT) and balanced fast‐field echo (B‐FFE) magnetic resonance (MR) datasets

	Intensity	
Structure	CT	B‐FFE MR
Prostate	50	360
Seed	2050	85
Background	0	400

## C. NMI algorithm

Mutual information registration algorithms use voxel intensity values to evaluate information content in a pair (base and match) of images. The formulation of NMI implemented in the Analyze software that we used was described by Studholme and colleagues^(17)^ in 1999, and is given by Equation 1:

(1) $$NMI(A,B)=\frac{H(A)+H(B)}{H(A,B)}$$

It is defined as the ratio of the sum of marginal entropies *H*(*A*) and *H*(*B*) of images *A* (base) and *B* (match), and their joint entropy *H*(*A,B*) in the region of image overlap. These entropies measure the information content in images *A* and *B* and in their overlap region, respectively.

The registration process consists of two steps: transformation and fusion.^(18)^ During the transformation step, the algorithm performs trial translations and rotations (and possibly other operations such as scaling) of the match image so as to transform its coordinate system into the coordinate system of the base image. In the search process, the algorithm calculates the information content of each image and of the overlap region. It then uses those values to evaluate NMI in the transformed image. The algorithm carries out this process iteratively, comparing the new value with the old one until it finds a maximum value of NMI, which ideally should occur when the images are well matched. In this circumstance, the amount of shared information in the individual images is maximized—or equivalently, the information content in the combined image is minimized. The fusion step involves simultaneous display of the base and transformed match images in the base image coordinate system.

To register image datasets according to the NMI measure, we used Analyze 5.0, a commercially available software package that provides comprehensive, generic tools for visualization, processing, and quantitative analysis of biomedical images. In particular, the NMI registration module in Analyze 5.0 allows specification of registration search parameters such as volume of interest (VOI), intensity threshold, transformation search range, and so on. The registration process is performed using a progressive image resolution search approach in which translations and rotations become finer with subsequent iterations. Further details regarding algorithm implementation were presented by Camp and Robb at Medical Imaging 1999: Image Processing (Bellingham, WA, 1999).

## D. Feature lines method

**Figure 3:**
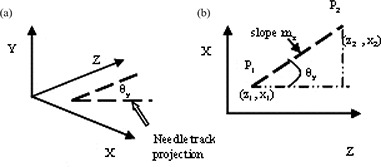
Orientation of a feature line: (a) rotation about the Y‐axis; and (b) $\theta_{y}$, angle of rotation in the X—Z plane can determined from a slope $m_{x}$ of the line joining points P1 and P2.

For implanted prostate glands, the X‐ and Y‐axis rotation angles required for image registration can, in theory, be determined from corresponding features in CT and MR datasets that run primarily in the Z direction. Suitable features might include visible needle tracks (MR) or seeds (CT), and their path in 3D space can be determined by finding the location of their centroids in the axial slices of the image volume. If features are identified as seed trains (CT) and needle tracks (MR), then associated feature lines can be obtained by fitting the points $\left(x_{i}(z_{i}),y_{i}(z_{i})\right)$ at the centroid locations to straight lines. Each feature line can then be expressed by a pair of equations with independent variable $z:x^{\text{fit}}=m_{x}z+b_{x}$ and $y^{\text{fit}}=m_{y}z+b_{y}$. Rotation angle $\theta_{y}$ (see Fig. 3), which represents the Y‐axis rotation of the feature line with respect to the Z direction in the imaging coordinate system, can be determined from the slope $m_{x}$, and similarly, rotation angle $\theta_{x}$ can be determined from the slope $m_{y}$ according to Equation 2:

(2) $$\theta_{y,x}^{CT,MR}=-\tan^{-1}(m_{x,y}^{CT,MR})$$

Applying a rotation $R_{y}(-\theta_{y}^{MR})$ to the MR (match) image volume will make a feature line in this volume parallel to the Y–Z imaging plane. A rotation $R_{y}(-\theta_{y}^{CT})$ will do likewise for the corresponding feature line in the CT (base) image volume. Subsequently applying a rotation $R_{x}(\Delta \theta_{x})$ to the MR volume, with $\Delta \theta_{x}$ determined according to Equation 3, will set the feature lines from the two modalities parallel to each other:

(3) $$\Delta \theta_{x}=\tan^{-1}\left(m_{y}^{CT}\right)-\tan^{-1}\left(m_{y}^{MR}\right)$$

Finally, applying a rotation $R(\theta_{y}^{CT})$ to both image volumes will maintain the feature lines parallel to each other, while restoring the CT volume to its original orientation. Therefore, the complete transformation (rotations only) that must be applied to points ${\vec{P}}^{MR}$ in the match volume to make a pair of corresponding feature lines in the base and match volumes parallel to each other is an ordered product of elementary rotations, as follows:

(4) $${\left({\vec{P}}^{MR}\right)}_{rot}=R_{y}(\theta_{y}^{CT})\times R_{x}(\Delta \theta_{x})\times R_{y}(-\theta_{y}^{MR})\times {\vec{P}}^{MR}$$

By applying this transformation to the match volume before NMI registration, we can eliminate X and Y rotations from the NMI transformation matrix, and so limit the second stage of the hybrid registration process to 4 DF (three translations and Z rotation only).

## E. Registration

### E.1. Simulated datasets

The simulated image data were designed to serve as test input to the Analyze 5.0 NMI algorithm and were created in the form of DICOM‐compatible files. We first attempted registration of simulated CT and MR datasets using a rigid‐body transformation and allowing 6 DF, with and without restriction on the transformation search parameters. The ranges of restricted X and Y translations were limited to 40% and 30% respectively of the image field of view. These limits were chosen based on the relative size of the simulated prostate in the image. The Z translation search range was not restricted, because the prostate volume extends through all the slices and such a restriction would not allow the algorithm to search through all meaningful information. The X, Y, and Z rotation ranges were limited to ±10 degrees, because we would not expect to see greater rotation in the clinical setting. Registration was also attempted allowing 4 DF (X, Y, Z translation and Z rotation) with the same restrictions imposed on search parameters. Datasets with and without Poisson noise added were processed to assess the algorithm's performance under such conditions.

The test procedure was as follows:

- The MR (match) image dataset was first translated by a known amount between 0.87 mm (3 voxels) and 3.48 mm (12 voxels), in either the positive or negative X, or positive or negative Y, direction.
- The translated image dataset was registered to the original untransformed CT (base) image dataset.
- The differences between applied translation values and translations obtained from the registration transformation matrix reported by Analyze were noted.

### E.2 Clinical datasets

To determine relative X and Y rotations in a pair of clinical CT and MR datasets, we first chose three pairs of corresponding feature lines (seed trains and corresponding needle tracks) in these volumes. We then determined the centroid locations in each axial slice of the seeds and associated needle tracks for each pair of feature lines. Centroid coordinates were determined using two different methods: visual inspection and automatic intensity peak finding.

For the first method, clinical image volumes were imported into Analyze 5.0, and the user visually picked the center of a seed or seed void in each slice using a cursor. The X and Y coordinates for each point were recorded. For the second method, the image volume was read into a Matlab program, and the user once again picked the center of a seed or seed void with a cursor to initiate the process. The program then used this point as the center of a 7×7‐pixel region of interest constructed for further processing and fitted a one‐dimensional Gaussian function to the intensity profile across the full width of the region of interest in both the X and Y directions. The location of the seed or seed void center in each slice was subsequently obtained as the coordinates of the extrema of the fitted Gaussians.

Although both methods used for finding seed or seed void centroids gave consistent results for CT volumes, the intensity peak finding algorithm did not work well for the MR image volumes. The reason, we believe, is that the needle tracks in the MR images have a broad—and not well‐defined—profile, resulting in a fairly wide Gaussian fit, which makes it difficult to automatically determine the true centroid location accurately. Considering that, for the CT volumes, centroid locations determined by the manual method were consistent with those determined by intensity peak finding, we were confident that the manual method would perform well for the MR volumes also. We therefore continued our analysis using manual centroid picking only. The X and Y coordinates determined by the manual method were plotted against their corresponding Z locations (taken to be at the centers of the slices), and the lines of best fit were found. Subsequently, Equations 2 and 3 were respectively used to calculate $\theta_{x}$ and $\theta_{y}$ for the CT and MR image volumes and $\Delta \theta_{x}$.

Using another Matlab program, elementary rotations were applied to the clinical MR datasets for the three patients according to Equation 4. Transformed match volumes were then imported into Analyze 5.0, where they were registered to corresponding base (CT) image volumes. The NMI registration was limited to 4 DF, and was performed on a VOI that included anterior rectum and muscle tissue adjacent to the prostate gland. The X and Y translation search ranges were limited to 20% of the image size, and the Z‐rotation search range was limited to ±10 degrees. The Z‐translation search range spanned the full extent of the image volume. These limits were found by experience to allow the most efficient and accurate registration of the clinical CT–MR volumes.

### E.3 Registration accuracy

To determine the accuracy of registration of the clinical datasets, we used measuring tools provided in Analyze 5.0 and calculated the average distance, $L_{\text{ave}}$, (Equation 5) between the X, Y, and Z coordinates $\left(c_{i}\right)$ of several matching points in each pair of clinical volumes:

(5) $$L_{ave}=\frac{1}{n}\sum_{i=1}^{n}\left|c_{i}^{MR}-c_{i}^{CT}\right|$$

We picked 7, 6, and 8 matching point pairs from the base (CT) and transformed (MR) volumes for patients A, B, and C respectively. The errors associated with manual picking of point coordinates were estimated to be 2 pixels (0.59 mm) in the X and Y directions and half a slice thickness (1.50 mm) in the Z direction. The error in $L_{\text{ave}}$ was calculated according to Equation 6:

(6) $$\Delta L_{ave}=\frac{1}{\sqrt{n}}*\sqrt{{\left(\delta c_{i}^{MR}\right)}^{2}+{\left(\delta c_{i}^{CT}\right)}^{2}}$$

A root‐mean‐squares (RMS) distance was then obtained by combining the X, Y, and Z values of $L_{\text{ave}}$ in quadrature. Choosing the same matching points and following the same procedure, we also calculated $L_{\text{ave}}$ and RMS values for image registrations performed independently by one of us (AA) with the Procrustes algorithm implemented in VariSeed 7.1 (Varian Medical Systems).

## III. RESULTS

### A. Simulated datasets

We investigated the ability of the NMI algorithm implemented in Analyze 5.0 to reliably perform automatic rigid‐body registration of the simulated CT and MR prostate implant datasets when 6 DF are allowed. Possibly because of the large difference between background intensities in the CT and B‐FFE MR datasets, the algorithm initially failed to attempt the registration of the two volumes.

To circumvent this problem, we inverted the intensities of structures in the B‐FFE MR datasets and were then able to proceed with registration. Inverted intensities had values of 40, 315, and 0 for the prostate, seeds, and background, respectively.

The algorithm did not perform well with or without imposition of restrictions on search parameters. In both cases, we found that, although average differences between applied and reported X and Y translations were lower than a clinically desirable tolerance of 1 mm, maximum differences exceeded this tolerance and were as high as ~8 mm. In addition, small rotations (~1.5 degrees) about the Z axis and Z translations of up to 7 mm were observed in some transformation matrices.

We proceeded to test NMI algorithm performance with X and Y axis rotations eliminated from the registration process (as they would be when determined by the feature lines method). We used the same procedure and applied the same restrictions on search parameters as for the 6 DF registration. We also repeated the registrations after adding Poisson noise to our datasets.

We found that maximum differences between applied and reported translations now fell within a clinically desirable tolerance of 1 mm for both noiseless and noisy images. Although transformation matrices for images without noise contained no Z translations, they did contain small Z translations (≤0.4 mm) for images with Poisson noise. No non‐negligible rotations about the Z‐axis were observed in either case. Table 2 summarizes the results of this exercise.

**Table 2:** Results of four‐degrees‐of‐freedom registration of simulated datasets with and without Poisson noise, where CT is computed tomography and B‐FFE MR is balanced fast‐field echo magnetic resonance

	CT/B‐FFE MR inverted (mm) No noise			CT/B‐FFE MR inverted (mm) Poisson noise		
Difference	ΔX	ΔY	ΔZ	ΔX	ΔY	ΔZ
Average	0.20	0.27	0.00	0.22	0.31	0.14
Maximum	0.50	0.88	0.00	0.60	0.91	0.31
Minimum	0.00	0.00	0.00	0.02	0.00	0.00

### B. Clinical datasets

**Figure 4:**
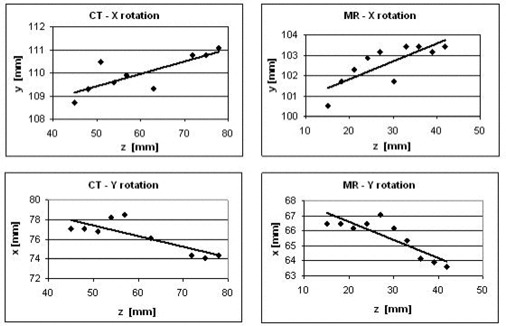
Sample plots used in graphical analysis for determination of $\theta_{x}$ (angle of rotation about X‐axis) and $\theta_{y}$ (angle of rotation about Y‐axis) for a pair of feature lines in a set of clinical image volumes for patient A.

Applying the feature lines method, we determined rotation angles $\theta_{x}$ and $\theta_{y}$ and the $\Delta \theta_{x}$ for three pairs of corresponding feature lines in the CT and MR image volumes for each of the three patients. The plots shown in Fig. 4 are examples of linear fits to CT and MR feature coordinates, obtained using the automated intensity peak finding method, for one pair of feature lines for patient A. Because of tissue flexibility and deformation subsequent to needle insertion into the prostate, we do not expect all the data points to closely follow a straight line; however, we do expect the points to follow an average direction that can be used to determine angles of rotation through graphical analysis. Table 3 reports average angle values determined from the results of three separate point selection trials for each pair of feature lines for patient A.

**Table 3:** Relative rotation angles for three pairs of feature lines in the image volumes for patient A, where ave denotes an average, CT is computed tomography, and MR is magnetic resonance

	$\theta_{y,\text{ave}}$ (degrees)		$\theta_{x,\text{ave}}$ (degrees)		$\Delta \theta_{x}$ (degrees)
Patient A	CT	MR	CT	MR	CT‐MR
Needle 1	6.42±0.00	−6.59±0.24	2.56±0.03	4.94±0.27	−2.38
Needle 2	1.83±0.01	2.01±0.07	7.62±0.01	10.13±0.15	−2.51
Needle 3	2.00±0.05	1.98±0.10	1.86±0.06	3.31±0.14	−1.45
				$\Delta \theta_{x,\text{ave}}$	−2.11
				σ	0.58

**Figure 5:**
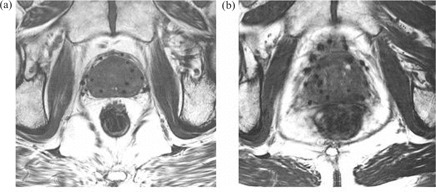
Clinical balanced fast‐field echo axial magnetic resonance slices of the prostate near mid‐gland illustrating the effect on image quality of motion artifact, (a) Patient A, no noticeable motion artifact present; image features appear sharp. (b) Patient B, considerable amount of motion artifact present; image features appear blurred.

With sequential application of $-\theta_{y}^{MR},\Delta \theta_{x},\;and\;\theta_{y}^{CT}$ rotations to the MR image volume, we parallelized MR feature lines with corresponding CT feature lines, and then followed with a 4 DF registration using the NMI algorithm. This hybrid approach successfully registered all three clinical image volume pairs, as judged by visual inspection—provided that some anterior bony structures and anterior rectum were included in the VOI. The presence of motion artifacts in the MR volume for patient B (see Fig. 5) required a VOI that extended beyond mid‐rectum posteriorly.

### C. Error analysis

The overall accuracy of registration of the CT and B‐FFE MR volumes was estimated by visual inspection to be ~1.5 mm for all three patients. Table 4 summarizes the results of the linear average distance calculations for the hybrid‐method image registrations performed for patients A, B, and C. Table 5 compares RMS values calculated for volumes independently registered by the hybrid and Procrustes algorithms.

**Table 4:** Average distances between X, Y, and Z coordinates of corresponding points (seeds or seed voids) in clinical computed tomography (CT) and magnetic resonance (MR) image volumes registered by the hybrid algorithm, where ave denotes an average

	$L_{\text{ave}}$ (mm)			$\Delta L_{\text{ave}}$ (mm)		
	X	Y	Z	X	Y	Z
Patient A	0.3	0.5	0.6	0.3	0.3	0.8
Patient B	1.1	1.4	1.0	0.3	0.3	0.9
Patient C	0.6	0.6	0.9	0.3	0.3	0.8

**Table 5:** Comparison of root‐mean‐squares (RMS) distances for clinical volumes registered by the hybrid and Procrustes algorithms

	${\text{RMS}}_{\text{hybrid}}$ (mm)	${\text{RMS}}_{\text{Proc}}$ (mm)
Patient A	0.8±0.6	1.3±0.6
Patient B	2.1±0.7	2.2±0.8
Patient C	1.3±0.7	1.4±0.6

## IV. DISCUSSION

Post‐implant dosimetry plays an important role in the assessment of prostate implant quality and correlates with treatment outcome. No single imaging modality provides the optimal visualization of both the implanted seeds and the soft tissue structures that is necessary for accurate calculation of dosimetric indices. However, registration of post‐implant CT and MR image volumes combines the necessary information in a fused image set and is therefore a promising approach for improving post‐implant dose estimation accuracy.

As compared with the T2‐weighted sequence, the B‐FFE MR image acquisition sequence used in the present work provided clinical images with better visualization of needle tracks. In addition, the contrast between the intensities of the structures of interest (prostate, bladder, rectum, seed voids, and other structures in the pelvic area) was better in the B‐FFE image volumes because intensity levels were higher than those in the T2‐weighted images. The better contrast is reflected in the higher information content in the B‐FFE images (for patient A, H(B−FFE)=6.91 and H(T2)=4.91 in the axial slices on average), likely making those images more suitable for registration using a voxel intensity–based algorithm such as NMI.

Our work indicates that the standard NMI algorithm implemented in Analyze 5.0 is not capable of reliable automatic rigid‐body registration of either simulated or clinical CT–MR datasets when 6 DF are allowed. We hypothesized that this result was due in large part to poor image resolution in the Z direction, which renders the algorithm insensitive to small rotations of the Y–Z and X–Z image planes, and thereby prevents accurate determination of X‐axis and Y‐axis rotation angles. The ultimate result is poor registration. Our results for 4 DF registration of simulated datasets suggests that the presence of Poisson noise in the images does not interfere with the ability of the NMI algorithm to successfully perform 4 DF registration within a clinically desirable tolerance of 1 mm.

We investigated the capability of a novel hybrid algorithm to perform semiautomatic 3D rigid‐body registration of post‐implant CT and MR prostate image volumes. The method consists of two steps. First, the feature lines method is used to achieve parallelization of corresponding features running primarily in the Z direction in complementary CT and MR image volumes. Parallelization of corresponding feature lines involves determining their relative rotations in the X–Z and Y–Z imaging planes, and subsequently applying those rotations to the MR (match) image volume. With this step completed, we effectively eliminate 2 DF from the NMI registration step that follows. The second step involves applying the NMI algorithm from Analyze 5.0 to parallelized CT and MR volumes.

We observed that the efficacy of 4 DF registration of parallelized CT and B‐FFE MR prostate implant image volumes depends on the extent of the VOI and the limits imposed on search parameter ranges. Restricting the transformation parameter search to a small range improves the algorithm's efficiency and helps prevent it from finding local maxima in the cost function. The hybrid algorithm was not able to register volumes with a VOI limited to prostate only. As McLaughlin et al. reported in 2004, we found that the VOI needs to include some anterior bony structures and the anterior rectum.

The presence of motion artifacts, such as were observed in the MR images for one patient (patient B), seems to hinder the NMI algorithm's ability to achieve good registration and, for patient B, necessitated a VOI that extended beyond mid‐rectum posteriorly. As well, rectum filling in the same patient was noticeably different between the CT and MR scans. This factor may have affected registration quality because of the possibility for greater change in prostate position between the two scans. It should also be noted that the results obtained here with the NMI algorithm in the Analyze 5.0 software package may differ from those for other NMI registration algorithms. In particular, the Analyze implementation makes use of image data sampling to build the joint entropy histogram and therefore may not fully utilize all of the information available in the registration VOI.

## V. CONCLUSION

A novel hybrid algorithm described in this paper shows promise in providing efficient and accurate 6 DF rigid‐body registration of CT and B‐FFE MR post‐implant prostate image volumes. Compared with the Procrustes algorithm, the new algorithm achieves registration with comparable accuracy, as demonstrated by RMS distance measures (Table 5). The Procrustes method requires approximately 20 minutes of dedicated user time, but the hybrid algorithm could be streamlined by automation of the feature parallelization process to provide semiautomatic registration in much less time. For the hybrid algorithm to achieve registration with acceptable transformation error, the registration VOI may need to include anterior rectum and some pubic bone. As well, care should be taken to minimize unnecessary movement during imaging to avoid motion artifacts that can reduce the accuracy of registration.

## ACKNOWLEDGMENTS

The present work was sponsored in part by an Alberta Cancer Board Translational Research Training in Cancer Fellowship and a Natural Sciences and Engineering Research Council of Canada PGS A Scholarship. We would like to thank Dr. Don Robinson for his generous help with CT‐scanning techniques, and the referees for their constructive comments.
